# Supplementary material for: The Specific Gravity-Free Method for the Isolation of Circulating Tumor KRAS Mutant DNA and Exosome in Colorectal Cancer
Source: Micromachines (Basel). 2021 Aug 20;12(8):987. doi: 10.3390/mi12080987 (PMC8400105; doi:10.3390/mi12080987)
Supplement: Supplementary file 1 [file micromachines-12-00987-s001.zip › micromachines-1314909-supplementary.pdf]

Supplementary Information

The Specific gravity-free method for Isolation of Circulating tumor KRAS mutant DNA and exosome in colorectal cancer

Tae Hee Lee[†,,1], Eunsook Park[2], Young-gon Goh[1], Han Byul Lee[1], Woo Sun Rou[3], Hyuk Soo Eun[†,*,4]*

[1]Research Institute for Future Medical Science, Chungnam National University Sejong Hospital (CNUSH), Sejong, Republic of Korea

[2]Department of Chemistry, Korea Advanced Institute of Science and Technology (KAIST),

291 Daehak-ro, Daejeon, Republic of Korea

[3] Division of Gastroenterology and Hepatology, Department of Internal Medicine, Chungnam National University Sejong Hospital, Sejong, Republic of Korea

[4] Division of Gastroenterology and Hepatology, Department of Internal Medicine, Chungnam National University Hospital, 282, Daejeon, Republic of Korea

†Both authors contributed equally to this work
*Both authors contributed as corresponding authors

*Address all correspondence to:
Tae Hee Lee, MT. PhD
Research Institute for Future Medical Science,

Chungnam National University Sejong Hospital (CNUSH),

Bodeum 7ro 20, Sejong, 30099, Republic of Korea

email: taehee1155@gmail.com
phone: (82)10-7112-8577

Hyuk Soo Eun, MD. PhD.

Division of Gastroenterology and Hepatology,

Department of Internal Medicine, Chungnam National University Hospital,

282, Daejeon, Republic of Korea

email: liver@kaist.ac.kr, hyuksoo@cnuh.co.kr
phone: (82)10-3470-0767

Statistics

Various associations were determined by calculating Spearman correlation coefficient (r) between ctDNA and exosome. A p-value <0.05 was considered significant. All statistical analyses were made through SPSS Statistics 26 (SPSS, IL).

Supplementary Figure.

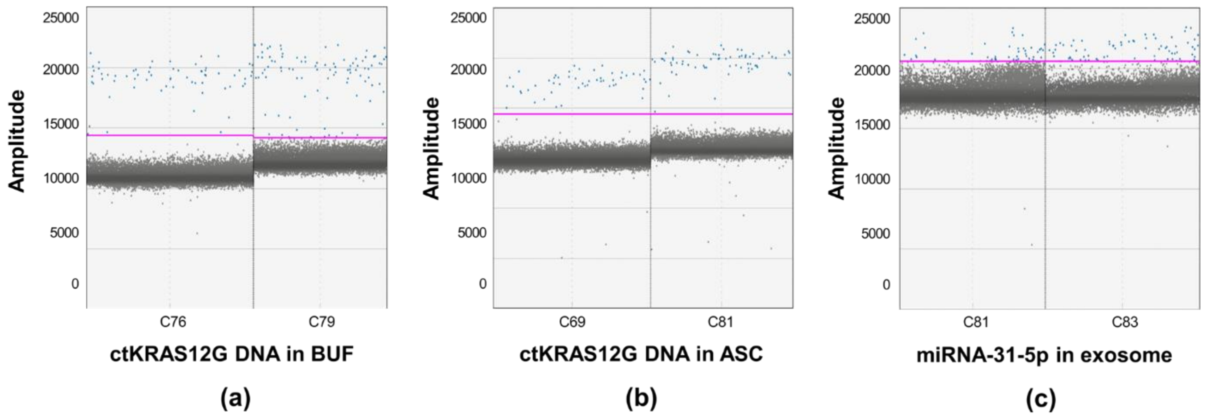

Figure S1. ddPCR analysis of (a) *KRAS* G12D mutation in buffy coat (blue: positive droplet), (b) *KRAS G12D* mutation in ascites (blue: positive droplet), and (c) miR-31-5 in exosome.

Supplementary Figure.

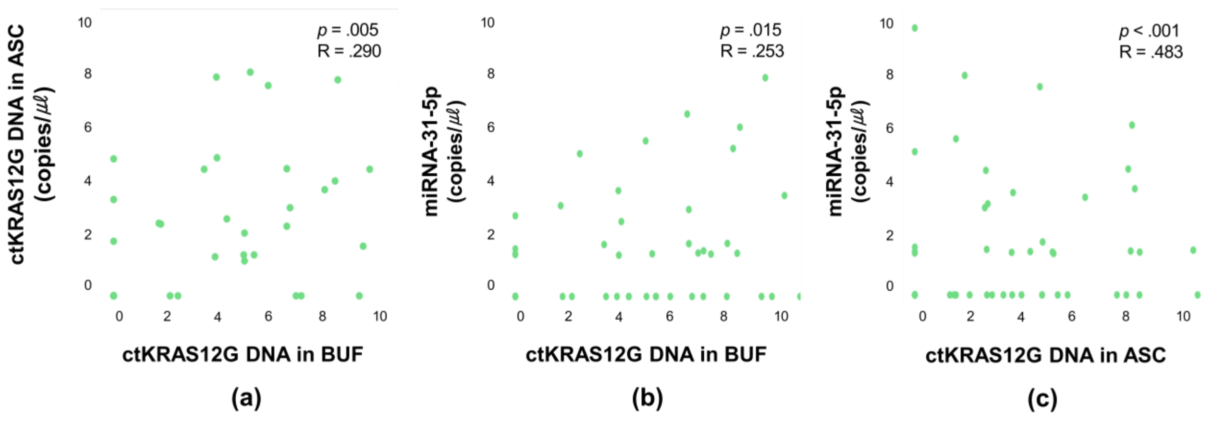

Figure S2. Spearman correlation analysis (a) between ascites and buffy coat of ctKRAS, (b) between ctKRAS in buffy coat and miR-31-5 in exosome, (c) between ctKRAS in ascites and miR-31-5 in exosome.

Supplementary Figure.

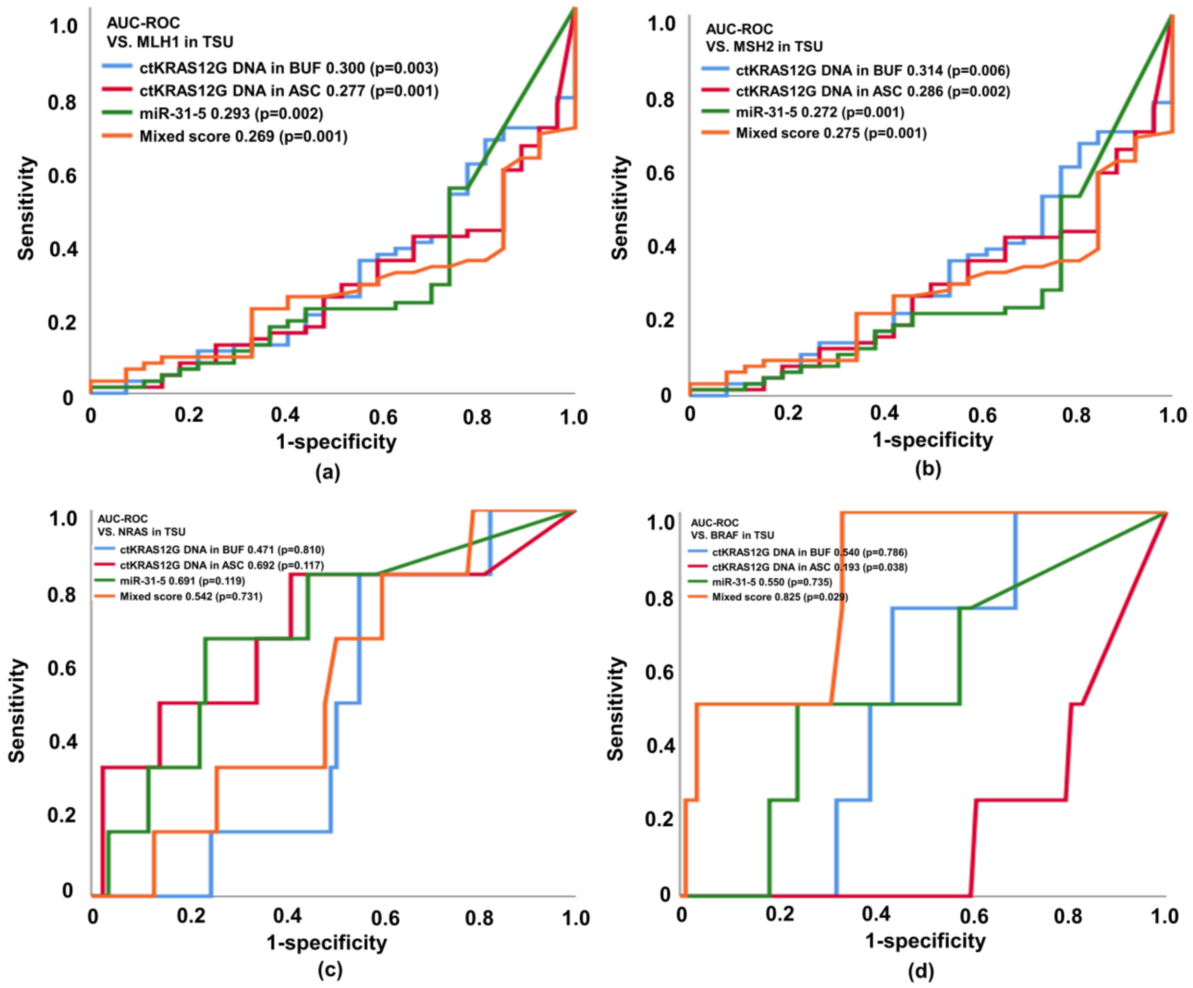

Figure S3. ctKRAS G12D mutation in buffy coat, ctKRAS G12D mutation in ascites, miR-31-5 in exosome, and mixed score reflects the pathological status of the tumor burden based on MLH1 (a), MSH2 (b), NRAS (c), and BRAF (d).

Supplementary Figure.

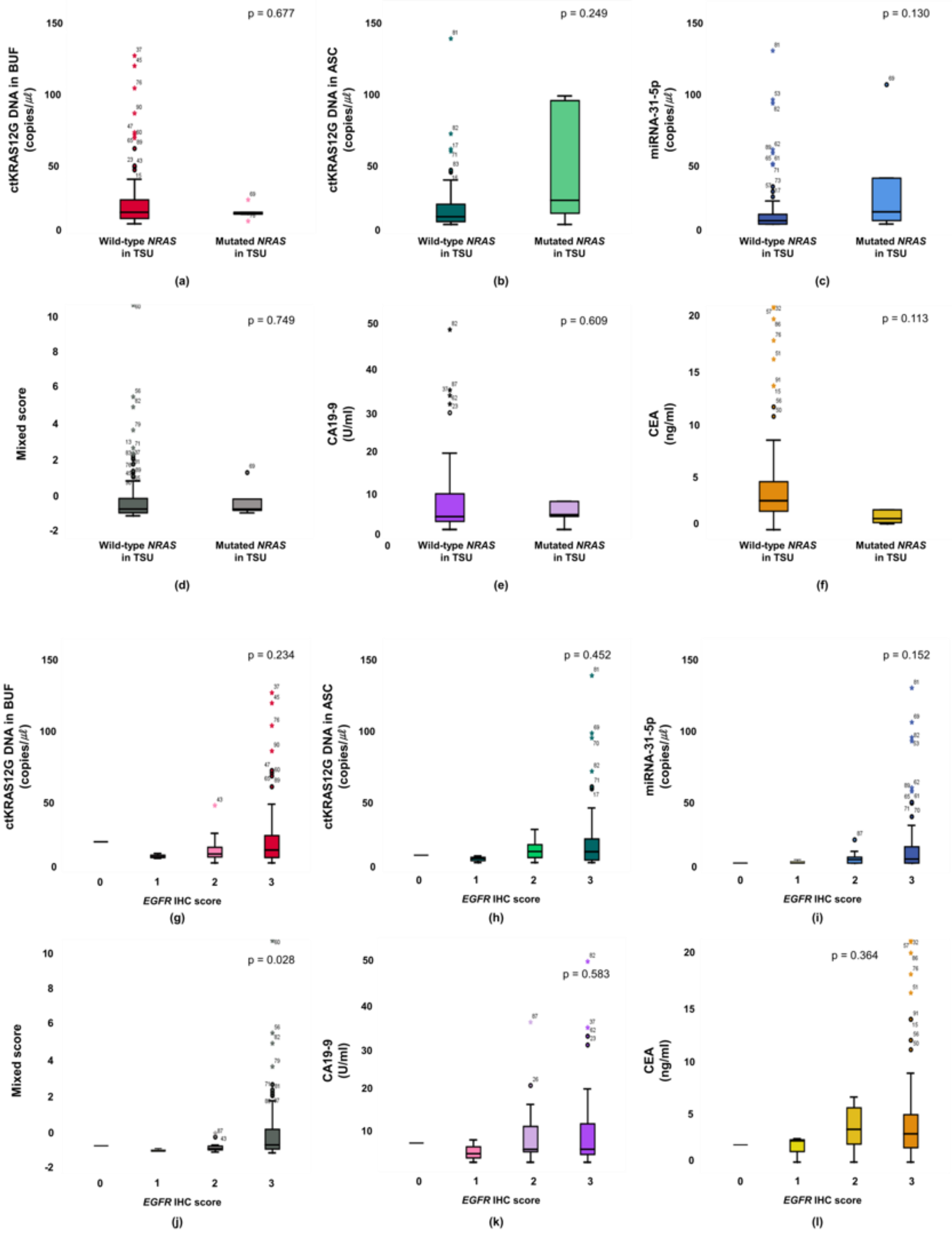

Figure S4. The expression levels in buffy coat (a), ascites (b), miR-31-5 (c), mixed score(d), CEA(e), and CA19-9(f) from mutant type and wild type of NRAS. The expression levels in buffy coat (g), ascites (h), miR-31-5 (i), mixed score(j), CEA(k), and CA19-9(l) from mutant type and wild type of EGFR.
